# Unusual Presentation of Disseminated Histoplasmosis in an Immunocompetent Host

**DOI:** 10.7759/cureus.44170

**Published:** 2023-08-26

**Authors:** Maya Subramanian, Ashin Mehta, Pinky Jha

**Affiliations:** 1 Medicine, Medical College of Wisconsin, Wauwatosa, USA; 2 Internal Medicine, Medical College of Wisconsin, Wauwatosa, USA

**Keywords:** disseminated histoplasmosis, histoplasma in immunocompetent patient, immunocompetent, disseminated, fungal lung infection, fungal, infectious disease pathology, infectious disease, adrenal histoplasmosis, histoplasmosis

## Abstract

Histoplasmosis, a fungal infection caused by the inhalation of *Histoplasma capsulatum *conidia spores, has been shown to cause disseminated disease in immunocompromised individuals. Disseminated histoplasmosis manifests as multi-system involvement including pulmonary and/or neurological disease. Imaging findings, such as pulmonary focal infiltrates, cavitary nodules, mediastinal, and hilar lymphadenopathy, are common. Here, we report a rare case of disseminated histoplasmosis in a 58-year-old immunocompetent male with no occupational exposure. This patient presented with primary adrenal insufficiency, and a subsequent CT-guided biopsy of the adrenal gland was performed and revealed numerous spores containing *Histoplasma capsulatum*. This patient also suffered from numerous pulmonary and neurological derangements, which are likely sequelae of the disseminated fungal infection. Ultimately, the patient succumbed to their illness and died. Preventing such outcomes relies on early detection and prompt management, which are crucial in treating disseminated histoplasmosis. Increased awareness of atypical presentations can enhance patient outcomes and alleviate the impact of this severe fungal infection. This case not only underscores the importance of considering disseminated histoplasmosis in an immunocompetent patient presenting with unexplained weight loss and adrenal insufficiency but also contributes to the limited literature on disseminated histoplasmosis in immunocompetent individuals.

## Introduction

Histoplasmosis is an endemic fungal infection caused by inhaling conidia of the fungus *Histoplasma capsulatum* [1]. The fungus was first described by Samuel Darling in 1906 and commonly presents with pulmonary symptoms. However, immunocompromised patients can present with disseminated disease [2-3]. Conidia of Histoplasma are found in soil containing high concentrations of bat or bird feces. Therefore, occupational exposure (farming, landscaping, construction) and cave exploration increase the risk of inhaling the fungus [4].

Although *Histoplasma capsulatum* is most prevalent in the Midwestern United States and Central America, an increasing number of cases have been reported around the world. The total number of reported cases in Asia is approaching 2000, with India, China, and Thailand leading the continent in incidence. However, the true worldwide prevalence of histoplasmosis is unclear and is often misdiagnosed as tuberculosis given the similarities in clinical presentation. In countries with a high prevalence of TB, patients with constitutional symptoms like weight loss and pulmonary symptoms frequently get treated for TB without considering the diagnosis of histoplasmosis [5].

Here, we present a case of disseminated histoplasmosis in an immune-competent individual without occupational exposure who initially presented with primary adrenal insufficiency.

## Case presentation

**Figure 1:**
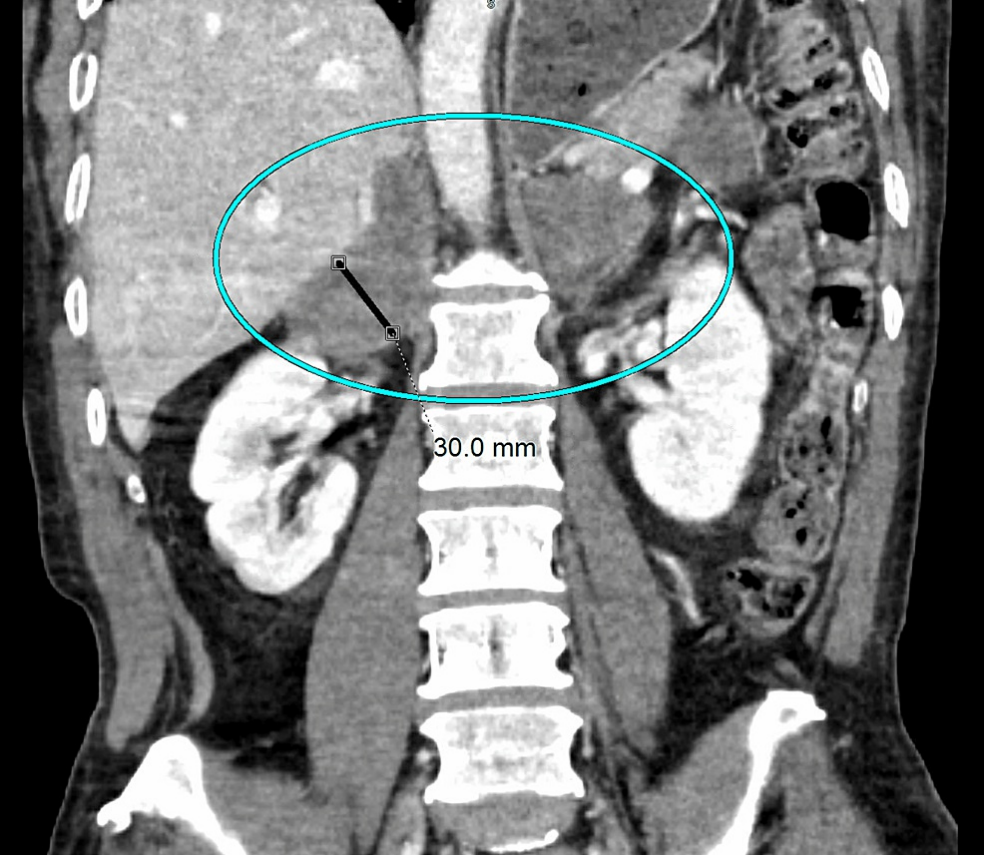
Bilaterally enlarged adrenal glands Diffuse thickening of the bilateral adrenal glands (cyan oval), of which the limbs measure up to 3 cm

A 58-year-old South Asian male without a significant past medical or smoking history presented with fatigue, weakness, syncope (fainting), and unintentional weight loss (80 lb.). The patient had immigrated to the midwestern United States from Bangladesh 30 years prior and reported frequent travel to Pakistan for IT-related work. The patient’s vitals and laboratory results were unremarkable except for hypotension (91/66 mmHg) and a potassium level of 3.2 mEq/L (reference level: 3.5-5.0 mEq/L). An abdominal CT scan revealed enlarged bilateral adrenal glands, with individual adrenal limbs measuring 30.0 mm with 60% washout (Figure 1). The expected size of the adrenal glands in an Asian adult male has been reported as 16.6 ± 3.6 mm [6].

**Figure 2:**
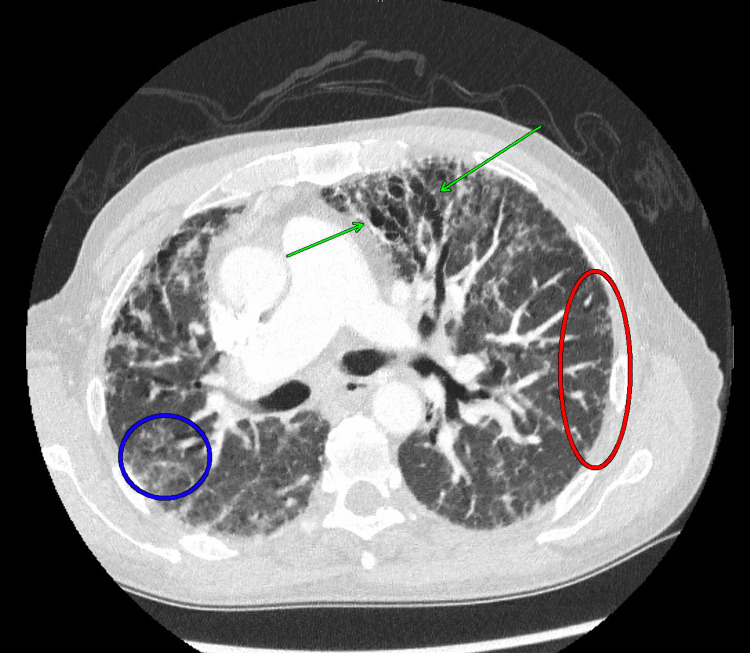
Diffuse traction bronchiectasis and interstitial reticulation Diffuse traction bronchiectasis was most pronounced anteriorly within the upper lobes and the right lower lobe (green arrows). This is accompanied by diffuse interstitial reticulation (red oval), both compatible with fibrotic change, and scattered ground-glass opacity (blue oval).

**Figure 3:**
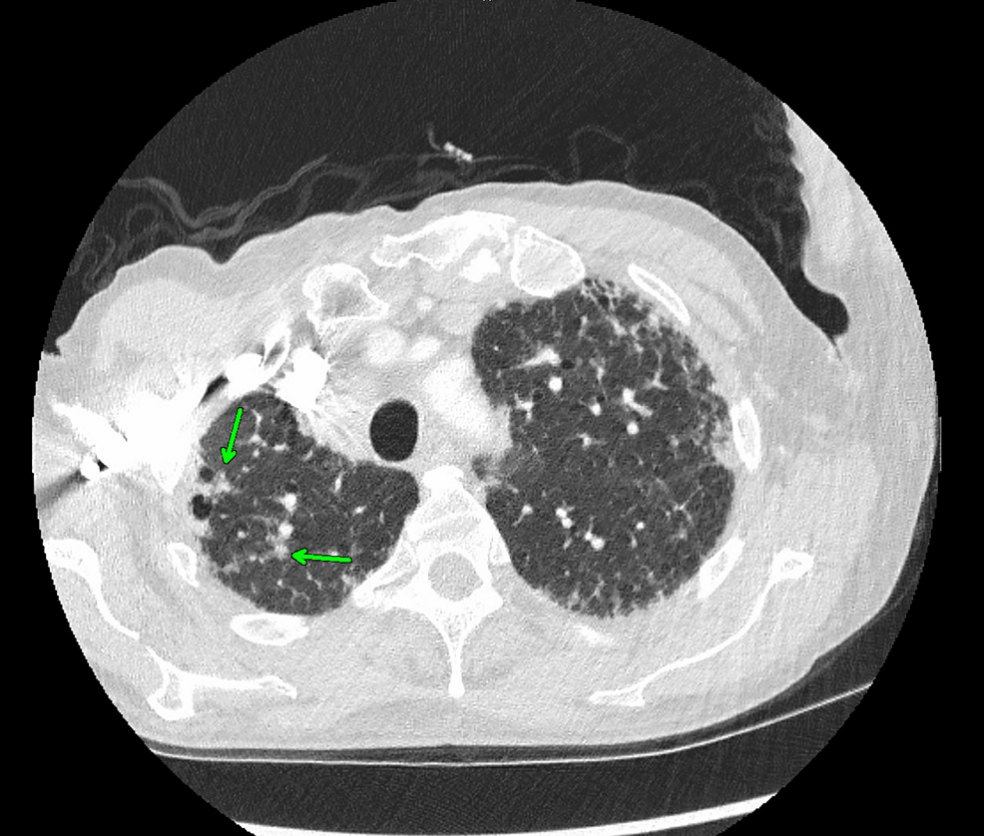
Upper lobe lung nodules

**Figure 4:**
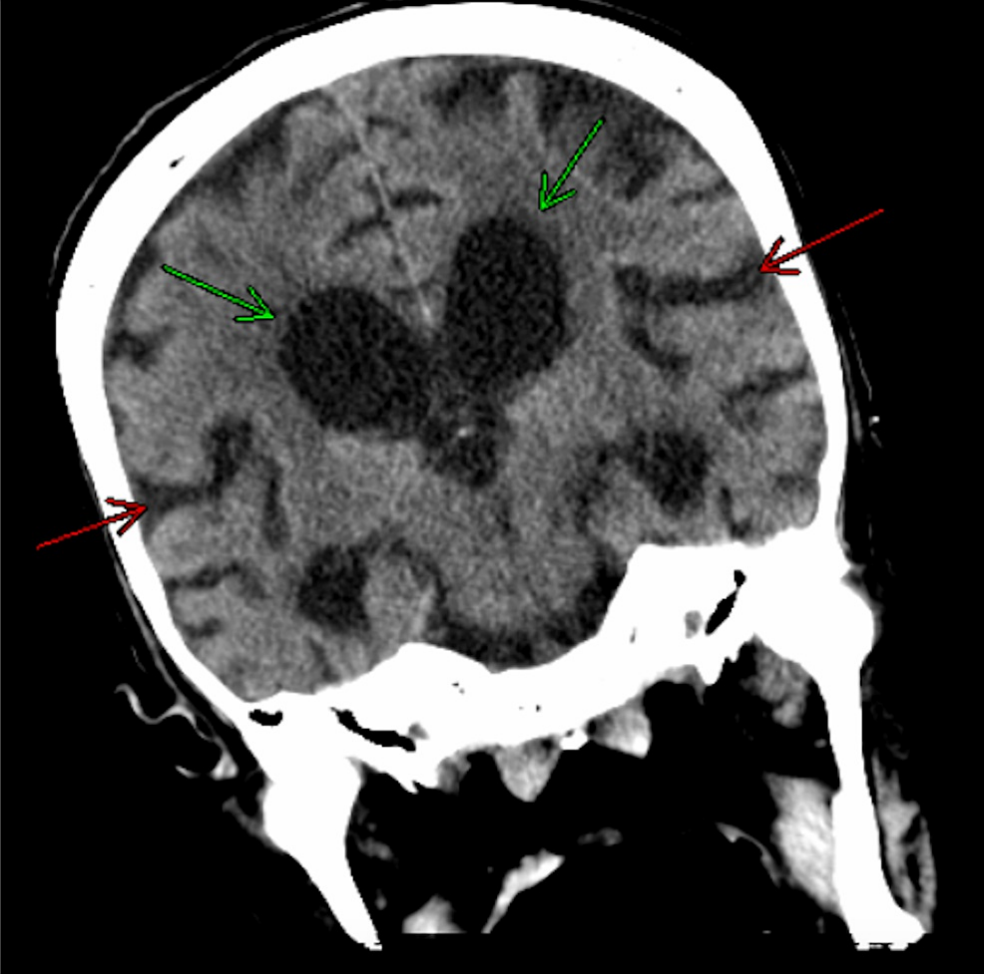
Ventriculomegaly and diffuse parenchymal volume loss Dilatation of the lateral ventricles (green arrows) and sulcal prominence (red arrows) on non-contrast CT of the head.

CT-guided biopsy of the adrenal gland was performed, and the pathology revealed numerous spores consistent with *Histoplasma capsulatum*. Chest CT revealed interstitial lung disease, fibrosis, and mediastinal adenopathy (Figures 2, 3). Subsequently, a head CT revealed generalized ventriculomegaly and parenchymal volume loss slightly more advanced for age (Figure 4).

While the patient’s pulmonary findings on chest CT could be attributed to histoplasmosis, the radiologist reported that it was nonspecific and could also be the result of environmental exposures, interstitial lung disease, systemic inflammatory disease, and medication-related toxicity. Extensive work was done that ruled out other causes like TB, HIV, and autoimmune diseases. Finally, given the clinical presentation, imaging, and biopsy findings, the patient was diagnosed with disseminated histoplasmosis. During his three-day-long inpatient stay, he was treated with liposomal amphotericin B at 3 mg/kg IV daily for two weeks and itraconazole 200 mg orally three times daily for three days. He was discharged with a prescription for daily 200 mg of oral itraconazole, which he was recommended to take for one year. The patient’s compliance with the treatment after discharge is not known.

Over four years, the patient continued to have respiratory and cognitive decline, resulting in multiple hospital admissions. Five years after his initial presentation, the patient was admitted to a Midwestern hospital where he was diagnosed with interstitial lung disease and COPD. A few months after his pulmonary diagnosis, the patient was admitted to the ED from a care facility with altered mental status, respiratory failure, and shock. During this admission, the patient was unable to communicate or follow commands. His response during a mental status evaluation was limited to intermittent eye-tracking. Vasopressors, stress dose steroids, and a high-flow nasal cannula were administered. After his blood pressure returned to baseline, he was transitioned to bilevel positive airway pressure (BiPAP). BiPAP-induced hypotension required further resuscitation with intravenous fluids.

Following stabilization, the patient was unable to tolerate a transition from IV to oral steroids. Oral steroids (hydrocortisone 10 mg) induced hypoxia and hypotension in the patient, who was unresponsive to IV fluids. Endocrinology was consulted and determined the patient had limited ability to metabolize oral medications. At the family’s decision, the patient's goal of care changed from treatment to comfort care. The patient passed away within a week of this transition.

## Discussion

Histoplasmosis is a systemic infection caused by the dimorphic fungal agent *Histoplasma capsulatum*. Diagnosis is based on clinical presentation supported by laboratory tests to rule out other possible causes. Although the infection is commonly diagnosed via blood or urine culture, a number of other tools can help clinicians confirm the diagnosis. A complement-fixation (CF) assay quantitatively measures histoplasmosis antibodies. Antibody titers of 1:8 to 1:16 are considered weakly positive, and titers of 1:32 or greater confirm active histoplasmosis [7]. Infection can also be confirmed with bronchoalveolar lavage and biopsies, of which culture and biopsy of the suspected site are crucial for diagnosis [8]. In our patient, a diagnosis was made after a biopsy of the adrenal glands that was positive for Histoplasma spores. Finally, imaging studies, such as CT scans and chest X-rays, can be used, as they help clinicians visualize structural changes caused by histoplasmosis [9].

The majority of immunocompetent individuals exposed to Histoplasma are asymptomatic. In those with symptoms, the spectrum of disease is determined by the load of inhaled spores [10]. If nonspecific lung defenses are unable to clear the fungus, bronchopneumonia occurs. During this time, macrophages ingest but are unable to kill the fungus. After 14 days, the adaptive immune response activates [11]. Common symptoms include fatigue, chills, headache, and body aches. Chest X-rays often display focal infiltrates, cavitary nodules, mediastinal, and hilar lymphadenopathy, but these findings are absent in 40-70% of cases [10]. Contrary to the typical presentation, our patient did not have respiratory symptoms. In addition, his initial chest CT was not suggestive of histoplasmosis. This case adds to the literature on histoplasmosis presenting without pulmonary findings.

Disseminated disease is driven by the failure of the cell-mediated immune response. These patients present with pathology in numerous organ systems [1,10,12]. GI manifestations include colonic ulcerations and polypoid masses, and CNS involvement results in patients suffering from meningitis, cerebral vasculitis, encephalitis, and focal brain lesions. One study found 70% of patients had GI manifestations at autopsy and CNS involvement is reported at a rate of 20% [12-13]. In addition to GI and CNS manifestations, disseminated disease can also cause skin lesions and endocarditis [14-15]. Presumably, the gradual cognitive decline our patient experienced could be the result of chronic histoplasmosis. However, the diffuse parenchymal loss and corresponding ventriculomegaly observed on the patient's head CT are consistent with normal age-related involution.

In contrast to the typical case of histoplasmosis, our patient first presented with adrenal insufficiency. Histoplasmosis-induced adrenal insufficiency is a rare complication believed to occur in less than 10% of cases with disseminated disease [13]. The adrenal glands are the most common organ outside the lungs involved if the host is immunocompetent, and males are at increased risk [16]. Adrenal involvement is caused by infected macrophages collecting in the adrenal sinusoids. Approximately 82% of disseminated histoplasmosis cases had adrenal involvement while 7% (live) or 11% (autopsy) cases showed Addison's disease [13]. It has been reported that histoplasmosis-induced adrenal insufficiency is associated with unintentional weight loss; our patient reported a decrease of 80 pounds [17].

Current treatment guidelines recommend itraconazole for at least 12 months along with a one to two-week course of amphotericin B in patients with disseminated disease [18]. Those with adrenal insufficiency should also receive corticosteroids [19]. The current literature supports the reversal of disseminated disease in some patients after prolonged antifungal therapy but true success rates are unclear. Our patient’s treatment followed guidelines but his outpatient adherence is unknown. Non-adherence could have contributed to his declining condition. We recommend management of disseminated histoplasmosis include strategies to ensure compliance. Such strategies consist of but are not limited to proper education and pre-scheduled follow-up appointments.

## Conclusions

What leads to the involvement of specific organ systems in disseminated histoplasmosis is unknown. What is clear is that isolated disease can present in any one or a combination of organ systems. For example, our patient presented with adrenal, pulmonary, and CNS involvement but did not develop skin, gastrointestinal tract, or cardiac disease. Clinicians must be aware of all clinical abnormalities associated with histoplasmosis, as early detection can greatly improve a patient’s prognosis. This case highlights the importance of the early diagnosis and treatment of disseminated histoplasmosis.

This case adds to the limited body of literature on disseminated histoplasmosis in immunocompetent hosts. We strongly encourage clinicians to consider the diagnosis of histoplasmosis in an immunocompetent patient presenting with unexplained weight loss and adrenal insufficiency. Despite treatment, this fungus can lead to severe disability and death. Therefore, steps to detect infection early and increase treatment adherence are critical in reducing mortality and morbidity.
